# Do Higher Transcranial Direct Current Stimulation Doses Lead to Greater Gains in Upper Limb Motor Function in Post-Stroke Patients?

**DOI:** 10.3390/ijerph20021279

**Published:** 2023-01-10

**Authors:** Raylene Pires, Adriana Baltar, Maria Paz Sanchez, Gabriel Barreto Antonino, Rodrigo Brito, Marina Berenguer-Rocha, Katia Monte-Silva

**Affiliations:** 1 Applied Neuroscience Laboratory, Department of Physical Therapy, Universidade Federal de Pernambuco, Recife 50670-900, Brazil; 2 NAPeN Network (Núcleo de Assistência e Pesquisa em Neuromodulação), Recife 55540-00, Brazil

**Keywords:** stroke, upper limb, transcranial direct current stimulation, stimulation parameters, dose–response

## Abstract

**Objective:** To investigate whether a higher number of transcranial direct current stimulation (tDCS) sessions results in a greater improvement in upper limb function in chronic post-stroke patients. **Materials and methods**: A randomized, sham-controlled, double-blind clinical trial was conducted in 57 chronic post-stroke patients (≥ 3 months after their injuries). The patients were allocated to receive sessions of tDCS combined with physiotherapy and divided into three groups (anodal, cathodal, and sham). The Fugl-Meyer Assessment of Upper Extremity (FMA-UE) was used to assess the sensorimotor impairment of the patients’ upper limbs before (baseline) and after five and ten sessions. The percentage of patients who achieved a clinically significant improvement (> five points on the FMA-UE) was also analyzed. **Results:** The FMA-UE score increased after five and ten sessions in both the anodal and cathodal tDCS groups, respectively, compared to the baseline. However, in the sham group, the FMA-UE score increased only after ten sessions. When compared to the sham group, the mean difference from the baseline after five sessions was higher in the anodal tDCS group. The percentage of individuals who achieved greater clinical improvement was higher in the stimulation groups than in the sham group and after ten sessions when compared to five sessions. **Conclusions:** Our results suggest that five tDCS sessions are sufficient to augment the effect of standard physiotherapy on upper limb function recovery in chronic post-stroke patients, and ten sessions resulted in greater gains.

## 1. Introduction

Stroke is one of the leading causes of physical disability among adults worldwide [1], with approximately 77% of survivors having chronic sensorimotor deficits that affect functional independence [2]. Often after a stroke upper extremity motor function is impaired, affecting patients’ daily living activities and quality of life [3]. Limb motor function is spontaneously recovered within six months [4,5], but rehabilitation can improve motor function even in the chronic phase [6]. However, motor function recovery through rehabilitation can be time-consuming and depends on plasticity [7,8]. Therefore, there is growing interest in tools that promote plasticity to enhance rehabilitation outcomes [9].

Transcranial direct current stimulation (tDCS) is a potential tool for increasing and accelerating cerebral cortex reorganization and facilitating motor learning by modulating cortical excitability [10]. After a stroke, tDCS promotes motor learning, known as priming, and can be used before or during motor training [11,12] to maximize upper limb motor gains [13]. Usually, anodal tDCS is applied to the motor cortex of the lesioned hemisphere to increase neuronal excitability, and cathodal tDCS in the non-lesioned hemisphere to decrease neuronal excitability [10,14,15,16,17].

Increasing evidence points to tDCS as an adjunctive treatment in post-stroke motor rehabilitation [18,19]. Despite promising evidence suggesting that tDCS potentializes stroke recovery [20,21,22], tDCS is not a one-size-fits-all treatment [23]. Indeed, studies have highlighted the variability in the effects of tDCS in stroke patients [24,25,26]. Understanding the factors affecting individual responses to electrical stimulation is crucial for identifying the optimal tDCS protocol to promote functional recovery after a stroke.

Several factors can influence the achievement of the best motor response with tDCS post-stroke, and questions still remain regarding the ideal dose of the number of sessions [23]. Indeed, randomized controlled trials (RCTs) that involved the use of intervention protocols with tDCS achieved significant improvements in upper limb (UL) motor function but with a large variation in the number of sessions [15,27,28,29,30,31,32,33,34,35]. These studies reported similar outcomes with five [15], ten [27,28,29,30,31], twelve [33,34], eighteen [34], and thirty-six sessions with the stimulation protocol [35], making it difficult to identify dose–response relationships. As such, our understanding of dose responses is limited.

To the best of our knowledge, no randomized clinical trial has investigated whether higher doses of tDCS produce greater clinical improvement in upper limb motor function. The dose of a non-pharmaceutical intervention is unclear, and inconsistent terminology can incorporate multiple dose dimensions, such as frequency, intensity, duration, and intervention length [36]. Here, the term dose is used to denote the dose dimension of the number of intervention sessions over time. Thus, we conducted a two-dose trial to compare the effect of the minimal number of sessions in studies with multiple sessions (five sessions) and the most common number of sessions among the studies (ten sessions). We hypothesized that higher doses of tDCS combined with physiotherapy would result in greater sensorimotor recovery of the upper limbs in chronic post-stroke patients. Additionally, we hypothesized a different dose–response relationship between the types of stimulation (cathodal vs. anodal tDCS).

## 2. Methods

### 2.1. Study Design

This double-blind, randomized, sham-controlled clinical trial was performed between November 2017 and January 2019 at the Laboratory of Applied Neuroscience (LANA), Universidade Federal de Pernambuco, Brazil.

All participants signed an informed consent form after being informed of the study objectives and procedures, which was performed in accordance with the Declaration of Helsinki. This study was approved by the local research ethics committee and was registered at www.clinicaltrials.gov (NCT03446378).

### 2.2. Study Population

Participants were recruited from a pre-existing list at the research laboratory via telephone. Patients of both sexes were included according to the following criteria: (i) diagnosis of hemorrhagic or ischemic stroke, confirmed by magnetic resonance imaging/computed tomography; (ii) ≥3 months after ictus; (iii) presence of sensorimotor sequelae in the upper limb; and (iv) age between 18 and 75 years. The exclusion criteria were as follows: (i) other neuromusculoskeletal injuries; (ii) cognitive deficits assessed by the Mini Mental State Examination (MMSE) ≤18 points [37]; (iii) contraindications for tDCS in stroke patients, such as the presence of skull defects, according to the safety protocols for its use [38]; (iv) performing physiotherapy elsewhere during the period of intervention; and (v) changes in medications that alter the excitability of the cortex and influence muscle tone in less than 3 months. In addition, to ensure the dose–response obtained by the intervention, the criteria for discontinuation of collection were adopted if: (i) there were more than two absences; and (ii) hemodynamic instability was present during the study.

### 2.3. Experimental Design

Randomization of patients who met the eligibility criteria was performed by an external researcher who was not part of any research process through the website www.randomization.com. This was stored on paper and kept in an opaque sealed envelope to keep the allocation confidential. Patients were randomized into three groups: (i) cathodal tDCS, (ii) anodal tDCS, and (iii) sham tDCS. The outcome evaluators were blinded to tDCS. A non-involved researcher was assigned to apply the tDCS. As such, this study was double-blinded.

**Figure 1:**
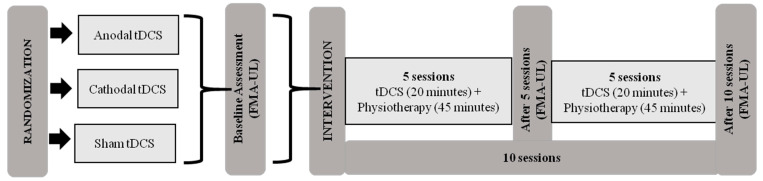
Intervention protocol. Abbreviations: tDCS, transcranial direct current stimulation; FMA-UE, Fugl-Meyer Assessment of Upper Extremity.

Initially, patients were evaluated using the Fugl-Meyer Assessment of Upper Extremity (FMA-UE) at pretreatment (baseline) and after 5 and 10 sessions. The Global Perception of Change Scale was also applied after starting treatment of five and ten sessions. The intervention protocol consisted of ten sessions of tDCS combined with physiotherapy for two consecutive weeks (5 days a week) (Figure 1).

### 2.4. tDCS Intervention

During the tDCS intervention (Neuroconn, Germany), two electrodes (5 × 7 cm) soaked in saline solution were applied to the scalp of the patients, applying a low-amplitude direct current (2 mA). The location of the primary motor cortex (M1) of the non-lesioned and lesioned hemispheres was based on the international 10/20 positioning system electroencephalogram. For the anodal tDCS group, the electrode was placed in the motor area ipsilateral to the lesioned hemisphere at points C3 or C4 (according to the 10/20 reference system), and the cathode was positioned over the contralateral supraorbital area. In the cathodal tDCS group, the cathode was positioned over the C3 or C4 motor area of the non-lesioned hemisphere, and the anode over the supraorbital area ipsilateral to the lesion. The sham tDCS group was subjected to the same procedure as the anodal tDCS group. The stimulation protocol lasted 20 min with a ramp up and down of 10 s, except for the sham tDCS group, which lasted 30 s to achieve a good level of blinding [39]. After each stimulation, a tDCS adverse effect questionnaire was completed [40]. Patients were then directed to perform physiotherapy sessions.

### 2.5. Physiotherapy Protocol

All patients, regardless of the stimulation group, underwent physiotherapy sessions. Each session consisted of kinesiotherapy for the rehabilitation of sensorimotor impairment of the upper limb, lasting 45 min. The kinesiotherapy protocol highlights 12 points following the neuroplasticity principles of Kleim and Jones [41]. These techniques included task-specific training, range of movement exercises, proprioceptive neuromuscular facilitation, and stretching. The tasks were progressively adapted to improve patient performance by targeting the main complaints and baseline results. The program was administered by a trained therapist.

#### Assessment Instruments

The outcome of the present study was related to impairment of upper limb motor function through the Fugl-Meyer Assessment of Upper Extremity (FMA-UE). The FMA-UE includes the motor domain and coordination/velocity. There is a total of 33 items, each of which contains an ordinal scale of three levels: (0) cannot be performed, (1) partially performed, and (2) performed completely, with a total score from 0 (complete hemiplegia) to 66 points (normal motor function) for the upper limb [42,43]. The Fugl-Meyer Assessment was performed at baseline (before intervention) and at the end of 5 and 10 intervention sessions.

### 2.6. Data Analyses

The normal distribution of data was analyzed using the Kolmogorov–Smirnov normality test. First, comparisons among the groups (anodal tDCS, cathodal tDCS, and sham tDCS) at baseline were performed according to the demographic and clinical characteristics of the samples. The chi-square test was used for categorical variables, and the Kruskal–Wallis and one-way ANOVA tests were used for categorical variables.

For the FMA-UE, repeated measurements for ANOVA 3 × 3 were calculated using the within-subject factor *time* (before and after 5 and 10 sessions) and among the factor *groups* (anodal, cathodal, and sham tDCS). Mauchly’s sphericity test was used to evaluate the validity of the sphericity assumption, and when necessary, was corrected using the Greenhouse–Geisser test. The paired *t*-test was used to compare the baseline and post-intervention assessments (baseline to after five sessions and baseline to after 10 sessions) in each group.

In addition, the number of patients who reached the minimal clinically important difference (mCID) of the FMA-UE was calculated at the end of 5 and 10 sessions. The assessment of patients’ mCID was considered as the minimum difference of five points obtained through FMA-UE for clinical improvement [44], which were categorized as: (i) no change (below five points), (ii) greatly improved (above five points), and (iii) very much improved (above ten points). For mCID analysis, a chi-square test was used to compare the differences among the groups in relation to the percentage of patients who reached a certain state based on evaluation of the scale.

All statistical analyses were performed using the statistical software IBM SPSS (Statistical Package for Social Sciences), version 23.0, UFPE, Recife, Brazil for Windows, with a significance level of 95% (*p* < 0.05).

## 3. Results

**Figure 2:**
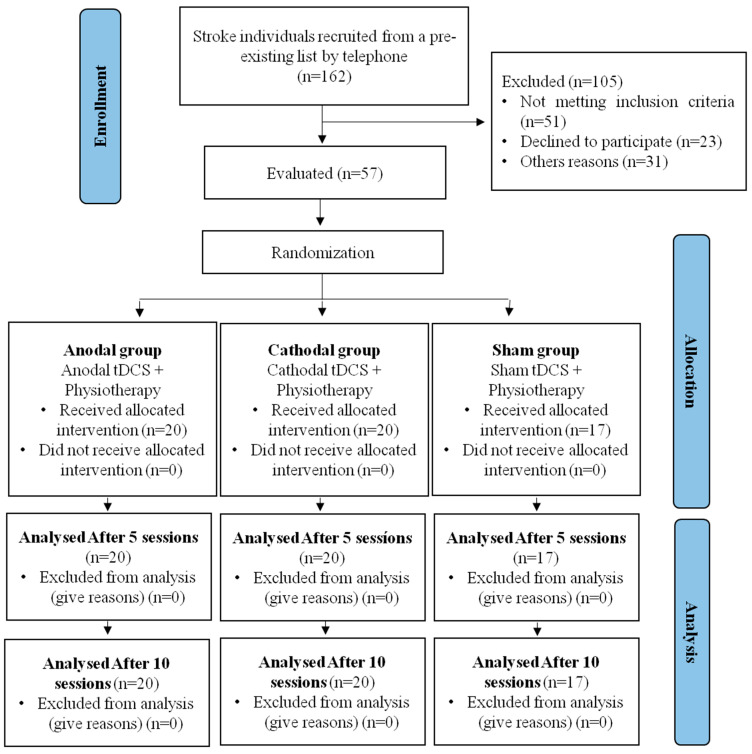
Study flowchart.

This study presents data collected from 57 participants with a post-stroke diagnosis, who were divided into three groups, as presented in the study flowchart (Figure 2). None of the participants reported serious adverse effects (hospitalization) during the tDCS stimulation. The only reported adverse effects were itching and tingling.

No significant differences were found among the anodal, cathodal, and sham tDCS groups in terms of the clinical and demographic characteristics of the study population (Table 1). Regarding the impairment of their upper limbs according to their FMA-UE scores, there was no significant difference among the groups at the baseline.

Repeated measures ANOVA revealed a significant effect for *time* (F = 56.504; *p* = 0.000) but not for *groups* (F = 0.013; *p* = 0.987) and *interactions* (F = 2.184; *p* = 0.089). Compared to the baseline, the FMA-UE score increased after five and ten sessions in the anodal and cathodal tDCS groups. However, in the sham group, the FMA-UE score increased after only ten sessions (Table 2).

**Figure 3:**
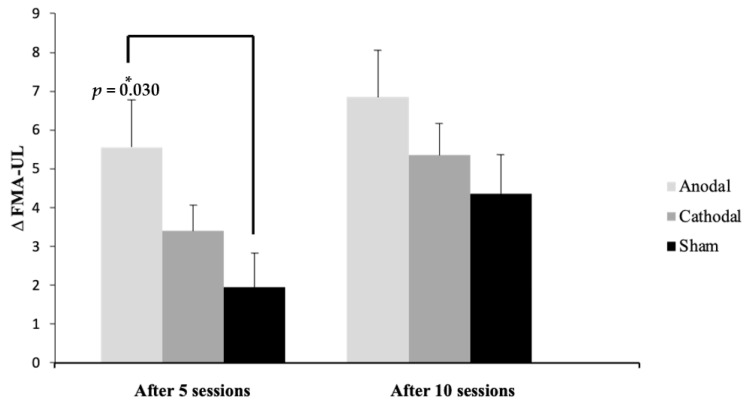
Mean and standard deviation (SD) of the ∆ (difference from baseline) of Fugl-Meyer Assessment of Upper Extremity (FMA-UE) scores after five and ten sessions compared to baseline between the groups, shown as the difference among the groups. * Significance: *p* ≤ 0.05 Tukey’s test.

Figure 3 presents the mean difference from the baseline after five and ten sessions in each group, showing a statistically significant difference (*p* = 0.03) between the anodal and sham groups after five sessions.

**Figure 4:**
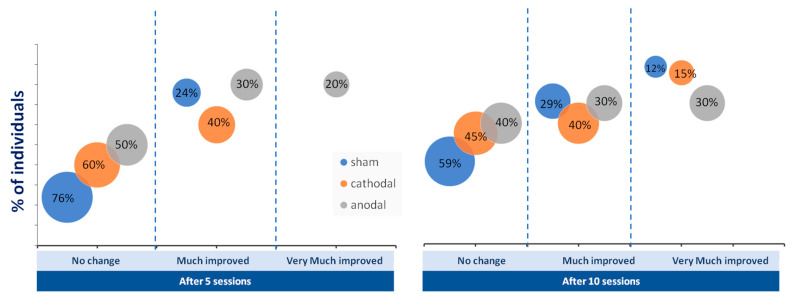
Percentage of individuals who achieved no change (below five points on Fugl-Meyer Assessment of Upper Extremity), much improvement (above five points), and very much improvement (above ten points) in upper limb motor function after five and 10 sessions of anodal (gray circles), cathodal (orange circles), and sham (blue circles) transcranial direct current stimulation (tDCS).

The number of patients who reached the mCID of the FMA-UE did not differ among the groups (*p* > 0,05; chi-square test). However, the percentage of people who achieved greatly improved and very much improved categorizations of FMA-UE was greater in the tDCS group than in the sham group after five and ten sessions (Figure 4).

## 4. Discussion

The results of the current study indicate that the applied tDCS protocols optimized the effect of physiotherapy by accelerating the response and achieving a greater effect size than with treatment alone for upper limb function recovery. After ten sessions, the patients who combined tDCS with standard physiotherapy, mainly anodal tDCS, had a higher percentage of clinically significant improvement in their upper limb motor function than the patients who did not receive the combination of therapies. In general, this result agrees with several systematic reviews with meta-analyses showing positive evidence for the effect of tDCS on upper limb motor function [45,46,47]. However, the wide variability in the number of tDCS sessions among the studies included in these reviews has prompted some questions—for example, the minimal number of sessions of tDCS required for optimal results, and whether a higher number of sessions results in greater gains.

Therefore, the current trial was designed to compare the effect size of five sessions (the minimal number of sessions in studies of multiple sessions) with ten sessions (the most common number of sessions among the studies) on upper limb function post-stroke recovery. Based on our findings, five tDCS sessions were sufficient to augment the effect size of physiotherapy on upper limb function recovery in chronic post-stroke patients. Previous research has demonstrated that both five and ten tDCS sessions can improve upper limb recovery.

In our study, mCID was achieved in all groups after five and ten sessions, but for a greater number of participants in the stimulation groups (anodal and cathodal) than in the sham group. These findings are consistent with a previous report that compared the mCID of three groups (anodal, cathodal, and sham) combined with a physiotherapy technique after 12 sessions, at a frequency of three times a week [26]. Taken together, both studies support the merit of combining physiotherapy with tDCS to augment clinical gains in stroke rehabilitation. However, the novelty of our study lies in the improvement of upper limb function after a small number of tDCS sessions (five sessions), suggesting that brain stimulation would also anticipate the response to conventional therapy. In 2021, a meta-analysis emerged that corroborated our findings, suggesting that a low number of sessions have a significant effect on upper limb rehabilitation [39]. Similarly, Zhang et al. found a peak in the efficacy of repetitive transcranial magnetic stimulation (rTMS) sessions on motor function after just five sessions [48]. However, to the best of our knowledge, none of these studies have investigated whether there is a dose–response relationship between the number of sessions. Thus, given the higher percentage of patients with greater improvements in the stimulation group after ten sessions compared to five sessions, our results seem to suggest a greater gain with higher doses.

It is important to mention that a meta-analysis [41] suggested that studies with the same stimulation parameters had a small non-significant potentiating effect size of tDCS in standard physical therapy for chronic upper limb hemiparesis [49,50,51]. Of the studies investigating the association between non-invasive brain stimulation and physiotherapy, the majority targeted the unaffected hemisphere with inhibitory stimulation, that is, cathodic tDCS [27,31], or low-frequency repetitive transcranial magnetic stimulation [52,53]. According to the latest theoretical model of the adaptive response of the brain after a stroke (bimodal balance recovery), the structural reserve after the brain is damaged should guide the understanding of the role of the unaffected hemisphere in functional recovery after stroke [54]. When the structural reserve is high, the suppression of the excitability of the hemisphere unaffected by the stroke could enhance recovery by reducing the interhemispheric inhibition of the stroke hemisphere [48]. However, when the structural reserve is low, the downregulation of the hyperexcitable unaffected hemisphere by brain simulation may not contribute to paretic upper limb motor ability and, in contrast, may be a greater contributor to limb paresis [54,55]. The unequivocal choice of stimulation protocol, irrespective of the level of the patient’s structural reserve, could reduce the clinical significance of non-invasive brain stimulation and may explain the discrepancy among studies.

The results of the present clinical trial showed that conventional therapy associated with anodal stimulation provided an improvement in upper limb function. These findings are in line with previous publications [29,31,35], suggesting that the activation of the affected hemisphere means the target can be relocated to more posterior [56,57] or more anterior regions in patients with more severe damage, predicting greater upper limb motor recovery after stroke [58].

Although there was homogeneity in the motor impairment level among our groups, a difference in patient stratification, regardless of the level of structural reserve, is likely to be the main limitation of our study. This could have influenced our result regarding the superiority of the anodal over the cathodal protocol. Nevertheless, in future studies, it may be interesting to compare the dose–response of both tDCS protocols in studies that choose stimulation protocols based on a neurophysiological evaluation of the level of the patient’s structural reserve. Another interesting direction for future work would be to compare the effect size of multiple tDCS sessions with clinical assessments in other domains of the International Classification of Functioning, Disability, and Health (ICF) [59], such as activity and participation.

A further limitation of this study is that the sample size was not calculated; therefore, as this is related to the power analysis, the data should be interpreted with caution. Finally, a further limitation of the study is the lack of a cut-off for FMA in patients with different motor impairment levels.

In summary, our preliminary evidence indicates that conventional therapy plus tDCS typically produces an earlier and greater effect than physical therapy alone, especially in anodal tDCS. Moreover, this clinical improvement is even faster and more effective with a minimum of five sessions, and as the number of sessions increases, clinical gains in upper limb function potentiate greatly.

## Figures and Tables

**Table 1:** Characterization of the study population.

Variables	Anodal tDCS (*n* = 20)	Cathodal tDCS (*n* = 20)	Sham tDCS (*n* = 17)	*p*-Value
**Age** *Years (Mean ± SD)*	58.25 ± 8.74	59.42 ± 9.96	56.81 ± 10.18	0.727 ^1^
**Sex** *Men n (%)*	8 (40.0%)	10 (50.0%)	6 (35.3%)	0.647 ^2^
**Dominance** *Right n (%)*	19 (95.0%)	17 (85.0%)	16 (94.1%)	0.199 ^2^
**Hemiparesis** *Right n (%)*	6 (30.0%)	11 (55.0%)	5 (29.4%)	0.199 ^2^
**Time Since Stroke** *Months (Mean ± SD)*	40.25 ± 36.29	31.00 ± 27.70	53.18 ± 38.10	0.197 ^3^
**FMA-UE Baseline** *Score (Mean ±SD)*	20.85 ± 14.91	21.60 ± 18.27	23.35 ± 18.26	0.948 ^3^

Abbreviations: SD, standard deviation; tDCS, transcranial direct current stimulation; UL, upper limb; FMA-UE, Fugl-Meyer Assessment of Upper Extremity. ^1^ ANOVA, one-way test; ^2^ Chi-square test (Fisher’s exact test); ^3^ Kruskal–Wallis test.

**Table 2:** Means and standard deviation (SD) of Fugl-Meyer Assessment of Upper Extremity (FMA-UE) score of each group at baseline, and after five and ten sessions.

	Anodal tDCS (*n* = 20)	Cathodal tDCS (*n* = 20)	Sham tDCS (*n* = 17)
**FMA-UE** *Score*			
*Baseline*	20.85 ± 14.91	21.60 ± 18.27	23.35 ± 18.26
*After five sessions*	**26.40 ± 16.49**	**25.00 ± 18.47**	25.17 ± 18.51
*After ten sessions*	**27.70 ± 16.29**	**26.85 ± 18.16**	**27.70 ± 19.17**

Numbers in bold indicate significant increase from baseline (*p* ≤ 0.05). Paired samples test. Abbreviation: tDCS = transcranial direct current stimulation.

## References

[1] Krishnamurthi R.V., Moran A.E., Feigin V.L., Barker-Collo S., Norrving B., Mensah G.A., Taylor S., Naghavi M., Forouzanfar M.H., Nguyen G. (2015). Stroke Prevalence, Mortality and Disability-Adjusted Life Years in Adults Aged 20–64 Years in 1990–2013: Data from the Global Burden of Disease 2013 Study. Neuroepidemiology.

[2] Lawrence E.S., Coshall C., Dundas R., Stewart J., Rudd A.G., Howard R., Wolfe C.D. (2001). Estimates of the prevalence of acute stroke impairments and disability in a multiethnic population. Stroke.

[3] Mayo N.E., Wood-Dauphinee S., Côté R., Durcan L., Carlton J. (2002). Activity, participation, and quality of life 6 months poststroke. Arch. Phys. Med. Rehabil..

[4] Stinear C. (2010). Prediction of recovery of motor function after stroke. Lancet Neurol..

[5] Stinear C.M., Barber P.A., Petoe M., Anwar S., Byblow W.D. (2012). The PREP algorithm predicts potential for upper limb recovery after stroke. Brain.

[6] Llorens R., Fuentes M.A., Borrego A., Latorre J., Alcañiz M., Colomer C., Noé E. (2021). Effectiveness of a combined transcranial direct current stimulation and virtual reality-based intervention on upper limb function in chronic individuals post-stroke with persistent severe hemiparesis: A randomized controlled trial. J. Neuroeng. Rehabil..

[7] Krakauer J.W. (2006). Motor learning: Its relevance to stroke recovery and neurorehabilitation. Curr. Opin. Neurol..

[8] Matthews P.M., Johansen-Berg H., Reddy H. (2004). Non-invasive mapping of brain functions and brain recovery: Applying lessons from cognitive neuroscience to neurorehabilitation. Restor. Neurol. Neurosci..

[9] Carey L.M. (2012). Stroke Rehabilitation: Insights from Neuroscience and Imaging.

[10] Bolognini N., Russo C., Souza Carneiro M.I., Nicotra A., Olgiati E., Spandri V., Agostoni E., Salmaggi A., Vallar G. (2020). Bi-hemispheric transcranial direct current stimulation for upper-limb hemiparesis in acute stroke: A randomized, double-blind, sham-controlled trial. Eur. J. Neurol..

[11] Stinear C.M., Barber P.A., Coxon J.P., Fleming M.K., Byblow W.D. (2008). Priming the motor system enhances the effects of upper limb therapy in chronic stroke. Brain.

[12] Stoykov M.E., Madhavan S. (2015). Motor priming in neurorehabilitation. J. Neurol. Phys. Ther..

[13] Murphy M.A., Willén C., Sunnerhagen K.S. (2011). Kinematic Variables Quantifying Upper-Extremity Performance After Stroke During Reaching and Drinking From a Glass. Neurorehabil. Neural Repair.

[14] Fregni F., Pascual-Leone A. (2007). Technology Insight: Noninvasive brain stimulation in neurology—Perspectives on the therapeutic potential of rTMS and tDCS. Nat. Clin. Pract. Neurol..

[15] Lindenberg R., Renga V., Zhu L.L., Nair D., Schlaug G. (2010). Bihemispheric brain stimulation facilitates motor recovery in chronic stroke patients. Neurology.

[16] Stagg C.J., Jayaram G., Pastor D., Kincses Z.T., Matthews P.M., Johansen-Berg H. (2011). Polarity and timing-dependent effects of transcranial direct current stimulation in explicit motor learning. Neuropsychologia.

[17] Nitsche M.A., Schauenburg A., Lang N., Liebetanz D., Exner C., Paulus W., Tergau F. (2003). Facilitation of implicit motor learning by weak transcranial direct current stimulation of the primary motor cortex in the human. J. Cogn. Neurosci..

[18] Bai X., Guo Z., He L., Ren L., McClure M.A., Mu Q. (2019). Different Therapeutic Effects of Transcranial Direct Current Stimulation on Upper and Lower Limb Recovery of Stroke Patients with Motor Dysfunction: A Meta-Analysis. Neural Plast..

[19] Orrù G., Conversano C., Hitchcott P.K., Gemignani A. (2020). Motor stroke recovery after tDCS: A systematic review. Rev. Neurosci..

[20] Butler A.J., Shuster M., O’Hara E., Hurley K., Middlebrooks D., Guilkey K. (2013). A meta-analysis of the efficacy of anodal transcranial direct current stimulation for upper limb motor recovery in stroke survivors. J. Hand Ther..

[21] Chhatbar P.Y., Ramakrishnan V., Kautz S., George M.S., Adams R.J., Feng W. (2016). Transcranial Direct Current Stimulation Post-Stroke Upper Extremity Motor Recovery Studies Exhibit a Dose–Response Relationship. Brain Stimul..

[22] Elsner B., Kugler J., Pohl M., Mehrholz J. (2020). Transcranial direct current stimulation (tDCS) for improving activities of daily living, and physical and cognitive functioning, in people after stroke. Cochrane Database Syst. Rev..

[23] Simonetta-Moreau M. (2014). Non-invasive brain stimulation (NIBS) and motor recovery after stroke. Ann. Phys. Rehabil. Med..

[24] Rossi C., Sallustio F., Di Legge S., Stanzione P., Koch G. (2013). Transcranial direct current stimulation of the affected hemisphere does not accelerate recovery of acute stroke patients. Eur. J. Neurol..

[25] López-Alonso V., Cheeran B., Río-Rodríguez D., Fernández-Del-Olmo M. (2014). Inter-individual variability in response to non-invasive brain stimulation paradigms. Brain Stimul..

[26] Tedesco Triccas L., Burridge J.H., Hughes A.M., Pickering R.M., Desikan M., Rothwell J.C., Verheyden G. (2016). Multiple sessions of transcranial direct current stimulation and upper extremity rehabilitation in stroke: A review and meta-analysis. Clin. Neurophysiol..

[27] Fusco A., Assenza F., Iosa M., Izzo S., Altavilla R., Paolucci S., Vernieri F. (2014). The ineffective role of cathodal tDCS in enhancing the functional motor outcomes in early phase of stroke rehabilitation: An experimental trial. Biomed. Res. Int..

[28] Ang K.K., Guan C., Phua K.S., Wang C., Zhao L., Teo W.P., Chen C., Ng Y.S., Chew E. (2015). Facilitating effects of transcranial direct current stimulation on motor imagery brain-computer interface with robotic feedback for stroke rehabilitation. Arch. Phys. Med. Rehabil..

[29] Kim J., Lee M., Yim J. (2019). A New Approach to Transcranial Direct Current Stimulation in Improving Cognitive Motor Learning and Hand Function with the Nintendo Switch in Stroke Survivors. Med. Sci. Monit..

[30] Jin M., Zhang Z., Bai Z., Fong K.N.K. (2019). Timing-dependent interaction effects of tDCS with mirror therapy on upper extremity motor recovery in patients with chronic stroke: A randomized controlled pilot study. J. Neurol. Sci..

[31] Yao X., Cui L., Wang J., Feng W., Bao Y., Xie Q. (2020). Effects of transcranial direct current stimulation with virtual reality on upper limb function in patients with ischemic stroke: A randomized controlled trial. J. Neuroeng. Rehabil..

[32] Rocha S., Silva E., Foerster Á., Wiesiolek C., Chagas A.P., Machado G., Baltar A., Monte-Silva K. (2016). The impact of transcranial direct current stimulation (tDCS) combined with modified constraint-induced movement therapy (mCIMT) on upper limb function in chronic stroke: A double-blind randomized controlled trial. Disabil. Rehabil..

[33] Beaulieu L.D., Blanchette A.K., Mercier C., Bernard-Larocque V., Milot M.H. (2019). Efficacy, safety, and tolerability of bilateral transcranial direct current stimulation combined to a resistance training program in chronic stroke survivors: A double-blind, randomized, placebo-controlled pilot study. Restor. Neurol. Neurosci..

[34] Triccas L.T., Burridge J.H., Hughes A., Verheyden G., Desikan M., Rothwell J. (2015). A double-blinded randomised controlled trial exploring the effect of anodal transcranial direct current stimulation and uni-lateral robot therapy for the impaired upper limb in sub-acute and chronic stroke. NeuroRehabilitation.

[35] Edwards D.J., Cortes M., Rykman-Peltz A., Chang J., Elder J., Thickbroom G., Mariman J.J., Gerber L.M., Oromendia C., Krebs H.I. (2019). Clinical improvement with intensive robot-assisted arm training in chronic stroke is unchanged by supplementary tDCS. Restor. Neurol. Neurosci..

[36] Hayward K.S., Kramer S.F., Dalton E.J., Hughes G.R., Brodtmann A., Churilov L., Cloud G., Corbett D., Jolliffe L., Kaffenberger T. (2021). Timing and Dose of Upper Limb Motor Intervention After Stroke: A Systematic Review. Stroke.

[37] Cockrell J.R., Folstein M.F. Mini-Mental State Examination. Principles and Practice of Geriatric Psychiatry. http://citeseerx.ist.psu.edu/viewdoc/download?doi=10.1.1.453.4452&rep=rep1&type=pdf#page=150.

[38] Bikson M., Grossman P., Thomas C., Zannou A.L., Jiang J., Adnan T., Mourdoukoutas A.P., Kronberg G., Truong D., Boggio P. (2016). Safety of Transcranial Direct Current Stimulation: Evidence Based Update 2016. Brain Stimul..

[39] Gandiga P.C., Hummel F.C., Cohen L.G. (2006). Transcranial DC stimulation (tDCS): A tool for double-blind sham-controlled clinical studies in brain stimulation. Clin. Neurophysiol..

[40] Brunoni A.R., Amadera J., Berbel B., Volz M.S., Rizzerio B.G., Fregni F. (2011). A systematic review on reporting and assessment of adverse effects associated with transcranial direct current stimulation. Int. J. Neuropsychopharmacol..

[41] Kleim J.A., Jones T.A. (2008). Principles of experience-dependent neural plasticity: Implications for rehabilitation after brain damage. J. Speech Lang. Hear. Res..

[42] Maki T., Quagliato E., Cacho E.W.A., Paz L.P.S., Nascimento N.H., Inoue M., Viana M.A. (2006). Estudo de confiabilidade da aplicação da escala de Fugl-Meyer no Brasil. Braz. J. Phys. Ther..

[43] Schiefelbein M.L., Salazar A.P., Marchese R.R., Rech K.D., Schifino G.P., Figueiredo C.S., Cimolin V., Pagnussat A.S. (2019). Upper-limb movement smoothness after stroke and its relationship with measures of body function/structure and activity–A cross-sectional study. J. Neurol. Sci..

[44] Page S.J., Fulk G.D., Boyne P. (2012). Clinically important differences for the upper-extremity Fugl-Meyer Scale in people with minimal to moderate impairment due to chronic stroke. Phys. Ther..

[45] Subramanian S.K., Prasanna S.S. (2018). Virtual Reality and Noninvasive Brain Stimulation in Stroke: How Effective Is Their Combination for Upper Limb Motor Improvement?—A Meta-Analysis. PM R.

[46] Van Hoornweder S., Vanderzande L., Bloemers E., Verstraelen S., Depestele S., Cuypers K., van Dun K., Strouwen C., Meesen R. (2021). The effects of transcranial direct current stimulation on upper-limb function post-stroke: A meta-analysis of multiple-session studies. Clin. Neurophysiol..

[47] O’Brien A.T., Bertolucci F., Torrealba-Acosta G., Huerta R., Fregni F., Thibaut A. (2018). Non-invasive brain stimulation for fine motor improvement after stroke: A meta-analysis. Eur. J. Neurol..

[48] Zhang L., Xing G., Fan Y., Guo Z., Chen H., Mu Q. (2017). Short- and Long-term Effects of Repetitive Transcranial Magnetic Stimulation on Upper Limb Motor Function after Stroke: A Systematic Review and Meta-Analysis. Clin. Rehabil..

[49] Kim D.Y., Lim J.Y., Kang E.K., You D.S., Oh M.K., Oh B.M., Paik N.J. (2010). Effect of transcranial direct current stimulation on motor recovery in patients with subacute stroke. Am. J. Phys. Med. Rehabil..

[50] Hesse S., Waldner A., Mehrholz J., Tomelleri C., Pohl M., Werner C. (2011). Combined transcranial direct current stimulation and robot-assisted arm training in subacute stroke patients: An exploratory, randomized multicenter trial. Neurorehabil. Neural Repair.

[51] Viana R.T., Laurentino G.E.C., Souza R.J.P., Fonseca J.B., Silva Filho E.M., Dias S.N., Teixeira-Salmela L.F., Monte-Silva K.K. (2014). Effects of the addition of transcranial direct current stimulation to virtual reality therapy after stroke: A pilot randomized controlled trial. NeuroRehabilitation.

[52] Galvão S.C.B., dos Santos R.B.C., dos Santos P.B., Cabral M.E., Monte-Silva K. (2014). Efficacy of Coupling Repetitive Transcranial Magnetic Stimulation and Physical Therapy to Reduce Upper-Limb Spasticity in Patients With Stroke: A Randomized Controlled Trial. Arch. Phys. Med. Rehabil..

[53] Seniów J., Bilik M., Leśniak M., Waldowski K., Iwański S., Członkowska A. (2012). Transcranial magnetic stimulation combined with physiotherapy in rehabilitation of poststroke hemiparesis: A randomized, double-blind, placebo-controlled study. Neurorehabil. Neural Repair.

[54] Di Pino G., Pellegrino G., Assenza G., Capone F., Ferreri F., Formica D., Ranieri F., Tombini M., Ziemann U., Rothwell J.C. (2014). Modulation of brain plasticity in stroke: A novel model for neurorehabilitation. Nat. Rev. Neurol..

[55] Kang N., Summers J.J., Cauraugh J.H. (2016). Transcranial direct current stimulation facilitates motor learning post-stroke: A systematic review and meta-analysis. J. Neurol. Neurosurg. Psychiatry.

[56] Pineiro R., Pendlebury S., Johansen-Berg H., Matthews P.M. (2001). Functional MRI detects posterior shifts in primary sensorimotor cortex activation after stroke: Evidence of local adaptive reorganization?. Stroke.

[57] Calautti C., Leroy F., Guincestre J.Y., Baron J.C. (2003). Displacement of primary sensorimotor cortex activation after subcortical stroke: A longitudinal PET study with clinical correlation. Neuroimage.

[58] Delvaux V., Alagona G., Gérard P., De Pasqua V., Pennisi G., de Noordhout A.M. (2003). Post-stroke reorganization of hand motor area: A 1-year prospective follow-up with focal transcranial magnetic stimulation. Clin. Neurophysiol..

[59] De Almeida P.M.D., Pereira C.S., dos Santos H.F.C.M., Martins A.C., Vital E., Noronha T., Costa R.J.D., Jacobsohn L., Caldas A.C. (2016). Categorização CIF de instrumentos de medida e intervenções utilizados na Fisioterapia em sujeitos com AVC. Cadernos de Saúde.

